# Teething as a Cause of Fever

**Published:** 1909-08-07

**Authors:** 


### Diseases of Children.

### TEETHING AS A CAUSE OF FEVER.

In the good old days teething was a useful explanation for many of the minor febrile attacks in infants, to which the medical attendant was indisposed to give a more definite diagnosis. In these iconoclastic times the tendency is towards another extreme, and it is by no means infrequent to meet with rational physicians who boldly assert that teething is a physiological process, and will not give rise to fever, except in the presence of some other pathological state. No doubt the true position lies more or less midway between these two extremes. We may grant that the process is physiological, but so are many others in which temporary or more serious disturbances occur. Menstruation, pregnancy and lactation are equally physiological, and are admitted to be at times deranged and the source of illness. Nevertheless it is important to be on one's guard in the case of febrile attacks in an infant who is teething. I have known such attacks to be diagnosed as the result of teething, although really due to gastro-enteric disturbance, paratyphoid and paracolon infections, acute anterior poliomyelitis, acute pyelitis and other causes.

An interesting case illustrative of some of these points was that of a healthy, well-nourished baby of the upper class, aged 16 months. She had already cut eight incisor teeth and two molars on the left side of her mouth, without any trouble of consequence. On June 19 she was distinctly feverish, ailing and fretful. Her temperature was 102° F., and nothing could be found to explain the attack except that she was cutting the two right molars. It could not be strictly maintained that there was any abnormal state of the mouth, nor were the gums unduly congested, tender or painful. The temperature jumped up and down, sometimes normal for a few hours and then running up again, or not quite falling to normal. On June 21, in the afternoon, the temperature rose to 104° F. Next day the upper molar had just penetrated the gum, and the temperature never rose to more than 100° F. On the following day it was normal, and the child apparently convalescent. In view, however, of the rapid approach of the lower tooth to the surface, the mother was warned that the fever would very probably return in a day or two. On June 25 the temperature rose to 101° F., and next day to 104° F. It ran the same kind of irregular course as before, and not until July 1 was there any evidence of anything further the matter with the child. On examining the mouth that day the gum over the erupting molar was distinctly a yellowish white, and its appearance had made the mother and nurse assume that the tooth was cut. On gently squeezing the gum one drop of quite sweet pus was obtained. It really seemed as if it were due to the necrosis of the superjacent cells of the mucous membrane. Naturally, with the evacuation of this, one expected the child was at the end of its trouble and that the temperature would fall. Next afternoon it was 104° F., and the lymph node at the right angle of the jaw was distinctly enlarged and tender. Here no doubt was the explanation of the further rise in temperature, namely, some slight septic absorption from the gum overlying the erupting tooth. The fever subsided in another two days and the child rapidly recovered. During the whole of this period of three weeks the amount of food taken was remarkably small, but the child wasted very little. Sleep was somewhat restless when there was much fever, but at other times was reasonably good. Beyond fretfulness and irritability there were no special nervous symptoms, and throughout there was no gastro-intestinal derangement. This was probably due to the fact that neither the nurse nor the doctor were anxious to press food on an unwilling and feverish infant. Most of the gastro-enteric troubles put down to teething are merely the result of over-feeding a feverish child, when its appetite is bad and the secretion of digestive juices limited or totally in abeyance.

It seems pretty obvious in the above case that the primary attack of fever was simply and entirely the result of teething, through setting up a slight local inflammatory reaction in the gum. Possibly the child was one of those who are liable to considerable fever on slight provocation, but it is rather remarkable that she had cut her previous teeth without definite trouble. She presented no sign or symptom of rickets. The subsequent fever may be said to have also been directly caused by the inflammatory process set up; in this case of sufficient severity actually to lead to the formation of pus, and sepsis for a day or two. The case may be taken as supporting both views of the effect of teething.

The treatment of these patients is a matter of much importance. Undoubtedly it is good policy not to adopt unduly active measures. Lancing the gums may do good by the local bleeding, but it certainly does not help in causing eruption of the tooth. No special antipyretic measures are needed, for the temperature rarely remains persistently high. The fever is merely a symptom and does little harm. The nurse should be instructed to keep the child lightly clad, the room well ventilated, and to give a warm bath or tepid sponging if the child is very restless and unable to sleep. On account of the liability to convulsions, it is generally advisable to prescribe a few grains of bromide, say iij-v., in one drachm of syr. mori, t.d.s., and to give hyd. c. cret gr. $\frac{1}{3}$-j. every night or on alternate nights. Here, again, one may mention the tendency of some doctors to speak of "the inevitable" grey powder. No doubt it is often given unnecessarily, but in my experience it never does harm in the doses here mentioned, and it is often followed by improvement. Certainly it cannot be regarded as the acme of good treatment to merely pay daily visits and watch the course of events. The parents are hardly likely to be satisfied, and though the doctor may be content that he is doing no harm, he must also realise that he is doing no good, and might as well wait until he is sent for to treat the convulsions or bowel derangement which may complicate the case.

